# Land Use and Land Cover Change Detection Using the Random Forest Approach: The Case of The Upper Blue Nile River Basin, Ethiopia

**DOI:** 10.1002/gch2.202300155

**Published:** 2023-09-17

**Authors:** Birhan Getachew Tikuye, Milos Rusnak, Busnur R. Manjunatha, Jithin Jose

**Affiliations:** ^1^ Department of Geography and Environmental Studies Debre Tabor University Debre Tabor P.O. Box 272 Ethiopia; ^2^ Institute of Geography Slovak Academy of Sciences Štefánikova 49 Bratislava 814 73 Slovakia; ^3^ Department of Marine Geology Mangalore University Mangalagangothri Karnataka 574199 India

**Keywords:** change detection, land use/land cover, landsat, random forest, upper Blue Nile River basin

## Abstract

Monitoring land use change dynamics is critical for tackling food security, climate change, and biodiversity loss on a global scale. This study is designed to classify land use and land cover in the upper Blue Nile River Basin (BNRB) using a random forest (RF) algorithm. The Landsat images for Landsat 45, Landsat 7, and Landsat 8 are used for classification purposes. The study area is classified into seven land use/land cover classes: cultivated lands, bare lands, built‐ups, forests, grazing lands, shrublands, and waterbodies. The accuracy of classified images is 83%, 85%, and 91% using the Kappa index of agreements. From 1983 to 2022 periods, cultivated lands and built‐up areas increased by 47541 and 1777 km2, respectively, at the expense of grazing lands, shrublands, and forests. Furthermore, the area of water bodies has increased by 662 km2 due to the construction of small and large‐scale irrigation and hydroelectric power generation dams. The main factors that determine agricultural land expansion are related to population growth. Therefore, land use and land cover change detection using a random forest is an important technique for multispectral satellite data classification to understand the optimal use of natural resources, conservation practices, and decision‐making for sustainable development.

## Graphical abstract

Random Forest‐based land use/cover classification is an effective way to classify satellite data. Cultivated land and built‐up areas significantly increased at the expense of forests, grasslands, and shrublands from 1983 to 2022 in the Blue Nile Basin. Water bodies also increased due to the construction of small and large dams for irrigation and hydroelectric power generation.

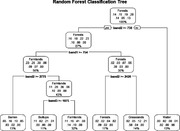

## 1 Introduction

Land cover mapping and monitoring are essential applications for Earth‐observing satellite sensor data used to assess ecosystem characteristics.^[^ ^1^ ^]^ The assessment of land use change at different spatial scales is necessary from many perspectives, including sustainable development,^[^ ^2^ ^]^ conservation and management of resources, land use planning,^[^ ^3^, ^4^ ^]^ humanitarian programs, as well as to climate change impact assessment and modelling.^[^ ^5^, ^6^, ^7^ ^]^ A quantitative understanding of land use change dynamics is critical for tackling food security, climate change, and biodiversity loss on a global scale.^[^ ^8^, ^9^, ^10^, ^11^ ^]^ Sustainable Development Goals (SDGs) have been achieved with the help of land cover (LC) information. The change in land use and land cover (LULC) has been one of the most noticeable changes to the earth's surface.^[^ ^8^ ^]^

Restoration and management of land resources can help combat climate change,^[^ ^12^ ^]^ protect biodiversity, maintain ecosystem services, and ensure the resilience of livelihoods.^[^ ^13^ ^]^ Therefore, assessing Land Cover is crucial, and is a fundamental theme of UN Global Geospatial Information Management (UN‐GGIM) on 14 fundamental geospatial data themes (UN‐GGIM).^[^ ^5^ ^]^ A variety of disciplines have benefited from remote sensing and GIS technologies, including geology, climatology, precision agriculture, crop yield, and soil nutrition prediction, contributing to the sustainable development of agriculture.^[^ ^14^ ^]^

LC information has greatly facilitated monitoring and achieving Sustainable Development Goals (SDGs).^[^ ^15^, ^16^ ^]^ In all countries, LC mapping needs to align with the global Land Cover Classification System (LCCS) for monitoring the SDGs.^[^ ^9^ ^]^ As a result, the LCCS offers an accurate and consistent assessment of land cover resources for developing LC products on various levels that are interoperable, scalable, and interchangeable. The FAO methodology has been perpetuated in many regions of the world at different levels and has been adapted to a wide range of fields.^[^ ^5^ ^]^

Understanding our changing land surface is essential to understanding climate change.^[^ ^8^, ^11^, ^17^ ^]^ Observations of vegetation and water from satellites provide data about changes in the earth's surface. Those satellites scan Earth's surface, revealing large‐scale and accurate information impossible to obtain from in situ ground‐level observations.^[^ ^18^, ^19^ ^]^ The data offer insight into land use, vegetation cover, and its changes, as well as hotspots prone to environmental issues.^[^ ^20^ ^]^ Through land monitoring with satellite data, our changing environment is mapped, allowing us to create mitigation plans for negative effects like desertification.^[^ ^8^, ^18^, ^20^, ^21^ ^]^

Multisource remote sensing and geographical data have increased in availability during the past four decades.^[^ ^22^ ^]^ Multisource data, as the name suggests, combines information from various sources. Examples include radar data, multispectral images from the Landsat satellite, hyperspectral airborne data, and geographical information like elevation and slope.^[^ ^21^, ^23^, ^24^ ^]^ To obtain the most details about the area being classified, it is preferable to use multisource data when classifying land cover from remote sensing data.^[^ ^25^, ^26^ ^]^ However, classifying multisource geographical and remote sensing data is a difficult challenge, particularly given that there is typically no appropriate multivariate statistical model for such data.^[^ ^26^, ^27^ ^]^ Multiple approaches have been used in remote sensing literature to address multi‐temporal classification issues. Multi‐temporal data can be classified by selecting key dates representing discriminative phenological stages.^[^ ^28^ ^]^

In the 21st century, images of the Earth's surface and algorithms for classifying them are widely available.^[^ ^29^, ^30^ ^]^ Research on LULC change detection is based on LULC classification.^[^ ^31^ ^]^ Traditional methods of visual interpretation and statistical analysis such as maximum likelihood, minimum distance, and spectral angles no longer meet LULC classification accuracy standards.^[^ ^32^, ^33^ ^]^ A challenge to these methods was the classification error between spectrally‐similar scenes, which prevented them from comparing more than a few scene covers.^[^ ^34^ ^]^ In supervised LULC classification, the most significant components are training data, classifiers, and supplemental datasets.^[^ ^17^, ^35^ ^]^ LULC data can be more accurately created by applying different LULC classification algorithms using modern tools (such as Google Earth Engine and ArcGIS Pro).^[^ ^11^, ^17^ ^]^ For instance, Support vector machines give the maximum classification accuracy because there are only a few difficult decision boundaries in the algorithm.^[^ ^17^, ^25^ ^]^ Thematic maps have been increasingly appealing to scholars as satellite sensor technology has made it possible to analyze land use and cover characteristics on the Earth's surface. The most intriguing agricultural applications include crop yield estimation and forecasting, soil moisture estimation, LULC change detection and mapping, and agricultural land classification.^[^ ^36^ ^]^

Machine learning algorithms have been employed since the launch of the first land observation satellite, Landsat‐1, in 1972 to classify pixels in Thematic Mapper (TM) images.^[^ ^11^, ^37^ ^]^ There are many methods to analyze a multispectral image. There are a variety of classification algorithms, including parametric traditional supervised methods like maximum likelihood and unsupervised methods, as well as non‐parametric machine learning techniques such as neural networks, support vector machines, decision trees, and combinations.^[^ ^38^, ^39^, ^40^, ^41^, ^42^, ^43^ ^]^ The neural networks are powerful, have an increased degree of strength and tolerance than conventional classifiers, and are a reasonable alternative to conventional classifiers. It is also suitable for classifying large and multispectral images.^[^ ^43^, ^44^ ^]^

More specifically, ensemble learning methods (bootstrap, boosting, etc.) have recently received a strong interest.^[^ ^11^, ^28^ ^]^ They consist in learning several weak classifiers to generate a classifier with a strong decision rule. A well‐known ensemble learning method is Random Forests (RF) which has demonstrated its ability to yield accurate land cover maps.^[^ ^45^, ^46^ ^]^ A more recent approach for classifying land covers has been based on ensemble methods, such as random forests (RF).^[^ ^47^ ^]^ RF is an ensemble learning algorithm based on the idea that a combination of bootstrap aggregated classifiers perform better than a single classifier.^[^ ^48^ ^]^ The RF algorithm is based on tree classifiers. The Random Forest grows a large number of classification trees. In addition to being one of the most accurate algorithms in the world. RF is also efficient to implement on large datasets, and its structure can be easily saved for future use.^[^ ^43^, ^49^, ^50^ ^]^

In general, agriculture is one of the most important economic activities in developing countries.^[^ ^51^, ^52^ ^]^ The upper Blue Nile River Basin (BNRB) is facing unparalleled changes in natural surroundings such as grasslands and forests due to the expansion of agricultural lands. This is to cope with the rapidly growing population and the growing demand for farming lands.^[^ ^52^, ^53^ ^]^ Therefore, this study is designed to use recently developed data mining methods for multispectral image classification in the BNRB. Hence, many of the previous studies^[^ ^54^, ^55^, ^56^, ^57^ ^]^ have employed traditional parametric statistics for the classification of multispectral imagery for land use mapping. In addition, they have applied classified satellite data to many environmental and socioeconomic applications. Hence, this study used the Random Forest approach for land cover classification and change detection of land use and land cover at the upper BNRB. As a result, this study aims to assess the robustness of random forest classification methods for providing accurate land cover maps over the upper Blue Nile River Basin using multispectral Landsat satellite imagery.

## 2 Experimental Section

### 2.1 Description of the Study Area

**Figure 1:**
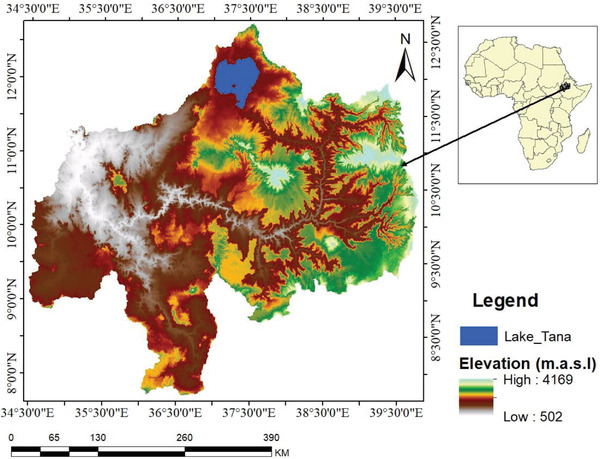
Study area map for the Abay Basin.

Geographically, the upper BNRB is located between 7^⁰^7′ N to 12^⁰^79′ N latitude and 34^⁰^49′ to 39^⁰^81′ E longitude (**Figure** **1**). The upper BNRB has the highest discharge volume, the second‐largest area,^[^ ^58^ ^]^ and the source of the Nile River is also the main tributary to the main Nile River not only to Ethiopia but also to the entire Nile River. There are several well‐known tributaries of the Blue Nile River in Ethiopia, including Beles, Gilgel Abay, Megech, Gumera, Ribb, Gulla, Dabus, Dinder, Rahad, Dura, Angar, Dedessa, Fincha, Wenchit, Chemoga, Muger, Jamma, Beshlo, Woleka.^[^ ^59^ ^]^ This river covers 17% of Ethiopia's land (176 000 km^2^ out of 1 100 000 km^2^)^[^ ^60^ ^]^ and has a mean annual discharge of 48.5 cubic kilometers (1912‐1997; 1536 m^3^ s^−1^). A large area of the central and southern Ethiopian Highlands is drained by this river.

Along the river's course through the central Ethiopian highlands, there are some places with gorges up to one kilometer deep.^[^ ^58^ ^]^ Blue Nile River is widely known for its long history and service to more than 238 million people in eleven nations.^[^ ^61^ ^]^ The Blue Nile River contributes 82% of the Nile flow along with the Tekeze and Baro‐Akobo Rivers. Several factors contribute to its decline, including population pressure in hydrologically sensitive areas, land degradation, upstream water diversion for irrigation and hydropower production, and changes in rainfall patterns (distribution, quantity, and timing). Water from the Nile River is the biggest source of life in Egypt and Sudan, so its availability is vital. The upper BNRB is the largest and most significant source of water in Ethiopia.^[^ ^62^ ^]^

Approximately 70% of the Basin's rainfall occurs from June to September, with an average of >2000 mm in the southwest and ≈1000 mm in the northeast.^[^ ^63^, ^64^, ^65^ ^]^ Approximately 1000 to 1800 mm of potential evapotranspiration occurs per year, but each is spatially variable.^[^ ^58^ ^]^ The mean annual temperature is ≈18.5 °C with a short seasonal variation of <3 °C, 17.7 °C in the summer season (locally known as *kiremt*, and 20.1 °C in the winter season (locally known as *Bega)*, respectively.^[^ ^66^ ^]^ Rainfall varied significantly between 1979 and 2014 over the western regions of the Blue Nile in the spring and southern areas in the summer. Similarly, to periodic variability in large‐scale circulation,^[^ ^67^ ^]^ regional variability is dominated by inter‐decadal signals.

### 2.2 Methods

#### 2.2.1 Image Processing

Geospatial technology has been applied to solve complex environmental problems at a local to Global scale in recent past years including all spheres of the earth, i.e., biosphere, hydrosphere, lithosphere, and atmosphere at different extents. In this case, remote sensors of various spatial resolutions provided information on vegetation, meteorological, oceanic, and hydrological types of information which will be used at different spatial extents.^[^ ^68^, ^69^, ^70^, ^71^ ^]^ Therefore, this research used satellite images acquired from Landsat 4–5 Thematic Mapper (TM) in 1983, Landsat 7 Enhanced Thematic Mapper Plus (ETM+) in 2003, and Landsat 8 Operational Land Imager (OLI) in 2022 which are stored in archives for 40 years, from 1983 to 2022.

During data downloading environmental considerations such as atmospheric conditions such as cloud cover, cloud shadows, relative humidity, soil moisture conditions as well as vegetation phenology were considered. During the downloading time, images taken from January to March, mostly dry season images, are downloaded. This is done to avoid confusion between vegetation and crops' spectral reflectance. Therefore, the maximum cloud cover for the scene is less than five percent during the downloading stages. The satellite data contained top‐of‐atmospheric reflections of the materials scanned on the earth's surface. Thus, converting the top of atmospheric reflectance to surface reflectance is a prerequisite for data extraction for different applications.^[^ ^72^, ^73^ ^]^

A mechanism for retrieving surface reflectance from the Top of the Atmosphere (TOA) is atmospheric correction. Despite this, estimating surface reflectance from the TOA satellite data requires an understanding of atmospheric conditions (e.g., water vapor and ozone) and aerosol contributions to atmospheric path radiance.^[^ ^74^ ^]^ In this way, atmospheric perturbations are removed, and the equivalent reflectance is given by objects on the ground surface, or surface reflectance (SR) at the Bottom of the Atmosphere (BOA).^[^ ^74^ ^]^ After atmospheric correction of the satellite data, composites of the band sets using median values were created. Next, the image has been mosaicked and sub‐set using the shape files for the research region.

#### 2.2.2 Image Classification

Seven information classes of interest to this study were chosen from the National Land Cover Database^[^ ^75^ ^]^ and also correspond to the CORINE land cover classes (https://www.eea.europa.eu/publications/COR0‐landcover) and supported by field observations based on dominant land cover types. CORINE land cover 2000 (CLC2000) also provides useful information about land cover types in 24 European countries information about land‐cover changes can be obtained from CLC data.^[^ ^76^ ^]^ Land use and Land cover (LULC) are now easier and faster to obtain than in the past due to advancements in remote sensing, geographic information systems, and machine learning technology.^[^ ^72^, ^74^ ^]^ Therefore, seven major LULC classes were considered for mapping the UBNB: bare lands, built‐ups, cultivated lands, grazing lands, shrub lands, forests, and water bodies (**Table** **1**).

**Table 1:** Description of LULC classifications classes.

LULC category	Descriptions
Bare lands	Areas devoid of vegetation, e.g., sediments, exposed rocks
Built‐ups	Settlements and roads
Cultivated lands	Irrigated agriculture area, agricultural fallow land, perennial crop
Grazing lands	Plant communities in which grasses are dominant, shrubs are rare and trees absent
Shrublands	Areas covered with woody vegetation mainly composed of shrubs less 5 tall
Forests	Land spanning more than 0.5 hectares with trees higher than 5 meters
Water bodies	Areas covered by perennial rivers, lakes, ponds, reservoirs,

The main goal of image classification is to categorize pixels from an image into land cover themes or classes. A single and best approach to image classification does not exist.^[^ ^77^ ^]^ The strategy that is employed relies on the type of data being analyzed, the available computer resources, and the planned use of the classified data. In terms of classification efficiency, random forest (RF) is among the best methods. Researchers from different backgrounds are drawn to it because of its inherent interdisciplinary characteristics.^[^ ^78^, ^79^, ^80^ ^]^ In contrast to traditional parametric multispectral image classification, the non‐parametric random forest algorithm was employed to classify Landsat imagery of the BNRB.

**Figure 2:**
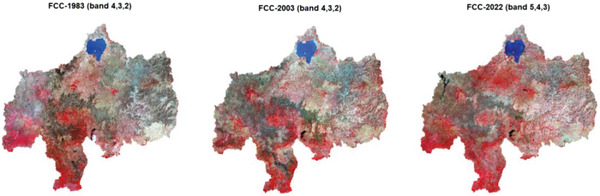
Upper Blue Nile River Basin false color composite bands for 1983, 2003, and 2022.

Multispectral band sets were used to advance the classification accuracy of the study area. Hence, blue, green, red, near‐infrared, short wave infrared one, and short‐wave infrared two band sets, as well as derived band sets, i.e., vegetation and water indexes, and ancillary data such as terrain and slope were applied to the classification. For instance, **Figure** **2** shows the Landsat 4–5, 7, and 8 false color composite (FCC), which is used to classify land use and land cover.

#### 2.2.3 Accuracy Assessment

The accuracy of the identified image was evaluated using randomly chosen samples of field‐collected ground truth points and Google Earth as a reference, and a confusion matrix analysis was carried out.^[^ ^81^ ^]^ To this effect, ground truthing was used to ascertain the accuracy of the LULC map. The mapped LULC was compared with observations on the ground. An error or confusion matrix is a common tool for describing thematic ambiguity in land‐cover data. Numerous measurements of agreement between data estimations and ground truth conditions are employed with this matrix.^[^ ^20^ ^]^

Therefore, an accuracy assessment is performed for 1983, 2003, and 2022 classified images using 200 randomly generated ground truths from Google Earth. Then, producer accuracy, user accuracy, and Kappa statistics were computed. The Kappa coefficient measures how much less error is produced by a classification procedure when compared to an entirely random classification.^[^ ^82^, ^83^, ^84^, ^85^ ^]^ indicated that a kappa value of < 40% is poor, 40%–55% is fair, 55%–70% is good, 70%–85% very good, and >85% excellent. Therefore, a confusion matrix has been computed to obtain the kappa index. Post‐classification was made to improve classification accuracy and reduce misclassifications. Thus, the results of RF classification were again improved and checked using visual analysis, reference data, and local knowledge.

#### 2.2.4 Change Detection

**Figure 3:**
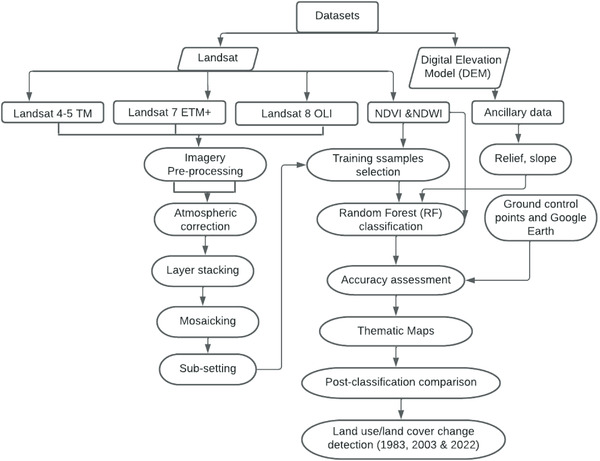
Flow chart of the adopted methodology.

In this section, LULC change is detected using comparative statistics. The percentage area for each land cover class was determined from the categorized image for each of the three years (1983, 2003, and 2022) individually using the post‐image classification process and ArcGIS software. Finally, it compares the area coverage of each land cover with time. **Figure** **3** indicates the flow chart of the adopted methodology for the study.

## 3 Results and Discussion

### 3.1 Accuracy Assessment

To assess UBNB classification accuracy, 200 Google Earth points were used each year. The 2022 land use and land cover maps had an overall accuracy of 96% and Kappa statistics of 91% (**Table** **2**). For the 2003 LULC, an overall accuracy of 93% and Kappa statistics of 85% were obtained, while the overall accuracy and Kappa statistics for the 1983 LULC for the basin were 91% and 83%, respectively (**Table** **3**). Therefore, according to the^[^ ^84^ ^]^ report, the kappa statistics obtained during the accuracy assessment stages are reliable, and the classified images can be used for further applications such as change detection, soil, and water conservation, policy recommendation, etc.

**Table 2:** Classification of Land Use and Land Cover Accuracy for 2022.

Class Value	WB	SL	GL	FR	BU	BL	CL	Total	User Accuracy	Kappa
WB	2.00	1.00	0.00	0.00	0.00	0.00	0.00	3.00	0.67	0.00
SL	0.00	41.00	0.00	1.00	0.00	0.00	0.00	42.00	0.98	0.00
GL	0.00	0.00	5.00	0.00	0.00	0.00	1.00	6.00	0.83	0.00
FL	0.00	0.00	0.00	6.00	0.00	0.00	0.00	6.00	1.00	0.00
BA	0.00	0.00	0.00	0.00	2.00	0.00	1.00	3.00	0.67	0.00
BL	0.00	1.00	0.00	0.00	0.00	2.00	0.00	3.00	0.67	0.00
CL	0.00	3.00	0.00	1.00	0.00	0.00	133.00	137.00	0.97	0.00
Total	2.00	46.00	5.00	8.00	2.00	2.00	135.00	200.00	0.00	0.00
Producer Accuracy	1.00	0.89	1.00	0.75	1.00	1.00	0.99	0.00	0.96	0.00
Kappa	0.00	0.00	0.00	0.00	0.00	0.00	0.00	0.00	0.00	0.91

NB: Water bodies (WB); Shrublands (SL); Grazing lands (GL); Forests (FR); Built‐ups; Bare lands (BL); Cultivated lands (CL)

**Table 3:** Accuracy assessment summary, 1983–2022, (%).

Land cover classes	1983		2003		2022	
	Producer's Accuracy	User's accuracy	Producer's accuracy	User's accuracy	Producer's accuracy	User's accuracy
Water bodies	100	100	100	100	100	67
Shrub lands	85	93	87	95	89	98
Grazing lands	62	71	63	71	100	83
Forest	75	100	75	100	75	100
Built‐ups	100	67	100	67	100	67
Bare lands	100	67	97	67	100	67
Cultivated lands	96	92	97	93	99	97
Overall Accuracy	91		93		96	
Kappa statistics	83		85		91	

### 3.2 Change Detection in Land Use and Land Cover

**Figure 4:**
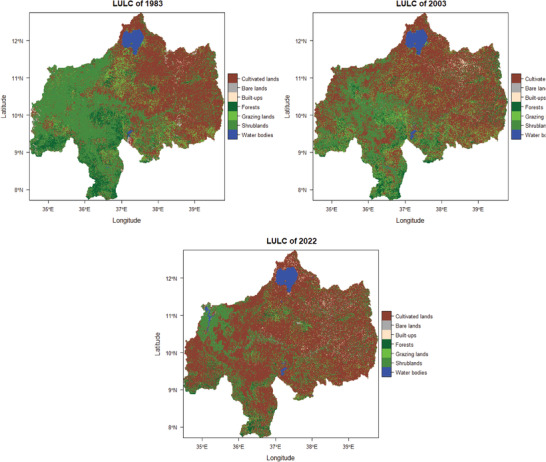
Upper Blue Nile River Basin land use/ land cover maps of 1983, 2003, and 2022.

For the three‐time slices, the study found seven different LULC classes. To supply the demand for food and fiber, agriculture has expanded all over the world at the expense of forests, savannahs, and steppes.^[^ ^51^ ^]^ The UBNB exhibits the same trend, with agriculture expanding during all analysis periods. The seven LULC classes that have been defined include water bodies, cultivated lands, bare lands, built‐up areas, forests, grazing lands, and shrub lands. **Figure** **4** displays maps of the UBNB's land cover for each of the three time periods.

**Figure 5:**
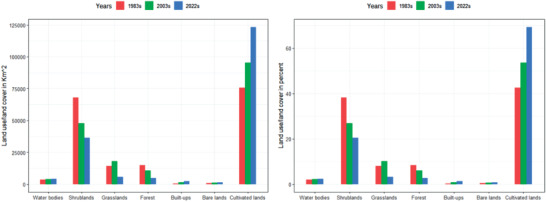
Upper Blue Nile River Basin land use/ land cover classes in square kilometers (km^2^) and percent (%) from 1983–2022.

**Figure** **5** for the UBNB shows the extent and rate of land cover change from grazing lands, shrublands, and forests into agricultural regions, urban areas, and water bodies. In 1983, the highest share of LULC from all classes was cultivated lands, which covered an area of 75 634 km^2^ and contributed 42.5% of the total area (Figure 5). The second highest share of LULC is shrublands, which have an area of 68 080 km^2^ (38.2%). Forest and grazing lands cover 14 257 km^2^ (8%) and 15 092 km^2^ (68.5%), respectively, whereas the area coverage of the built‐up area is 618 km^2^ (0.34%), bare lands are 882 km^2^ (0.5%), and water bodies are 3500 km^2^ (2%) of the total area of the UBNB.

**Figure 6:**
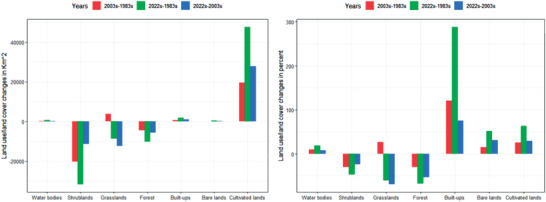
Upper Blue Nile River Basin land use/ land cover classes change in square kilometres (km^2^) and percent (%) from 1983–2022.

In 2003, the highest share of LULC from all classes was cultivated lands, which covered an area of 95 321 km^2^ and contributed 53.5% of the total area (**Figure** **6**). The second highest share of LULC is shrublands, which have an area of 47 864 km^2^ (26.8%). Forest and grazing lands cover 10 593 km^2^ (5.9%) and 18 071 km^2^ (10.2%), respectively, whereas the area of the built‐up area is 1363 km^2^ (0.8%), bare lands are 1013 km^2^ (0.6%), and water bodies are 3838.6 km^2^ (2.2%) of the total area of the UBNB.

In 2022, the highest share of LULC from all classes was cultivated lands, which covered an area of 123 175 km^2^ and contributed 69.2% of the total area (Figure 6). The second highest share of LULC is shrublands, which have an area of 36 419 km^2^ (20.5%). Forest and grazing lands cover 4954 km^2^ (2.8%) and 5627.3 km^2^ (3.2%), respectively, whereas the area coverage of the built‐up area is 2395 km^2^ (1.3%), bare lands are 1335 km^2^ (0.75%), and water bodies are 4162.2 km^2^ (2.3%) of the total area of the UBNB.

This demonstrates that in 2022, bushes, forests, and pastureland will only occupy 24.4% of the basin's total area. The remaining 69% of the area was arable land. It is clear from the statistics above that cultivated land expands at the expense of forests, grazing land, and other types of land. This encourages the use of marginal lands and hastens the degradation of land. From 1983 to 2022 time periods, cultivated lands and built‐ups increased by 47 541 km^2^ (63%) and 1777 km^2^ (288%) at the expense of grazing lands, shrublands, and forest lands (Figure 6).

## 4 Discussion

Multispectral sensors and geographical data have increased in availability during the past four decades.^[^ ^22^ ^]^ To obtain the most details about the area being classified, it is preferable to use multisource data when classifying land cover from remote sensing data.^[^ ^25^, ^26^ ^]^ These days, there is a large availability of satellites on the Earth's surface and classification algorithms.^[^ ^29^, ^30^ ^]^ There are a variety of classification algorithms, including parametric traditional supervised methods like maximum likelihood and unsupervised methods, as well as non‐parametric machine learning techniques such as neural networks, support vector machines, decision trees, and combinations.^[^ ^38^, ^39^ ^]^

The Random Forest classifier uses bootstrap aggregation to form an ensemble of classification and induction trees like tree classifiers.^[^ ^43^ ^]^ For instance^[^ ^11^ ^]^ indicated that machine learning models have been strongly recommended for the preparation and classification of land use and change detection maps. Random forests can also be very effective for estimating outcomes that are complex functions of predictors with many interactions or possibly a non‐linear function of the parameters.^[^ ^86^ ^]^ Furthermore,^[^ ^87^ ^]^ indicated that growing an ensemble of decision trees and allowing them to vote for the most popular class produced a significant increase in classification accuracy for land cover classification. On the other hand, the major disadvantage of RF is that measures of variable importance can be biased if the predictors are correlated. Studies have demonstrated the successful application of ensemble machine learning classifiers, such as Random Forests (RF), integrating remote sensing (satellite imagery) and ancillary spatial data, to improve the supervised classification accuracy of forest and other natural environment land cover maps.^[^ ^88^ ^]^

Thus, this study used the RF algorithm for the classification of multispectral Landsat 4–5, 7, and 8, which resulted in 81%, 85%, and 91% Kappa accuracy, respectively, for 1983, 2003, and 2022 LULC, which approves further use of the classified maps for LULC change detection and other research objectives. In line with this study,^[^ ^17^, ^78^, ^89^ ^]^ indicated that RF‐based classification of satellite data is used for land use, land cover classification, or agriculture classification. Furthermore, the use of multispectral bands such as blue, green, red, infrared, short wave infrared one, short wave infrared two, and derived bands such as vegetation and water indexes significantly improves the accuracy of the classification results. For instance,^[^ ^28^ ^]^ indicated that to help the classifier to learn the decision rule, features (also named variables and attributes) are used as input data in the classification system. The number and quality of input features are related to resulting accuracies, but also to computational time. Hundreds of spectral features, such as the NDVI (Normalized Difference Vegetation Index) for vegetation depiction, NDWI (Normalized Difference Water Index) for water detection, or NDBI (Normalized Difference Built‐up Index) for building detection, have been proposed and compared in remote sensing domains.

Random forest‐based land use and cover classification is an effective way to classify land cover because it uses a large number of decision trees to produce reliable results.^[^ ^25^, ^90^ ^]^ It also can handle large datasets and can be used to quickly produce accurate results. Additionally, it offers a better understanding of the spatial pattern of land use and cover. Key advantages of RF include their non‐parametric nature; high classification accuracy; and capability to determine variable importance. Another advantage of the random forest classifier is that it requires only two parameters to be set, whereas the SVMs require several user‐defined parameters.^[^ ^87^ ^]^ The relative value of various features throughout the classification process is also provided by this classifier, which is helpful for feature selection.^[^ ^91^ ^]^

The random forest classifier is less sensitive than other streamlined machine learning classifiers to the quality of training samples and overfitting.^[^ ^28^, ^45^, ^46^, ^48^ ^]^ also indicated that random forests have some advantages, such as a small training time and easy parameterization^[^ ^92^ ^]^ also indicated that Random Forest classification (RFC) has the advantages of high classification accuracy and the ability to measure variable importance in land‐cover mapping. RF provides an algorithm for estimating missing values; and flexibility to perform several types of data analysis, including regression, classification, survival analysis, and unsupervised learning.^[^ ^1^ ^]^ However, the split rules for classification are unknown, therefore, RF can be considered to be a black box type classifier.^[^ ^1^, ^48^ ^]^

In three time periods (i.e., 1983, 2003, and 2022), the dominant LULC of the UBNB was cultivated lands, followed by shrublands. Furthermore, cultivated land and shrublands alone occupied 80.73%, 80.3%, and 89.7% of the basin in 1983, 2003, and 2022, respectively. On the other hand, water bodies in the basin also showed increasing trends from 1983 to 2022. Hence, water bodies occupied 2%, 2.2%, and 2.3% of the basin in the 1983, 2003, and 2022 time periods, respectively. A major factor contributing to the development of water bodies in the study basin was the construction of small and large dams, both for irrigation purposes and hydroelectric power generation. For instance, water body areas increased by 662 km^2^ from 1983 to 2022 due to the construction of the Ribb Dam, Koga Dam, Megech River Dam, and Grand Ethiopian Renaissance Dam (GERD), and others in the BNRB. Koga Dam has a drainage area of 22 000 ha and a capacity to supply water for irrigating downstream farms of 7 000 ha.^[^ ^93^ ^]^ GERD is located on the Blue Nile in Ethiopia, ≈20 kilometers from the Ethiopian‐Sudanese border. There is a storage capacity of 74 BCM in the GERD reservoir. GERD creates a lake with an area of 1874 km^2^ that is spread over a distance of 246 km.^[^ ^94^ ^]^ The Ribb River is located in the Blue Nile basin, where it drains into Lake Tana. A 73‐meter‐high dam and a diversion weir 30 km downstream to irrigate 15 000 ha of land make up the Ribb River Dam.^[^ ^95^ ^]^

As a result, from the LULC analysis of three historical periods, it can be concluded that all periods indicated a decline in grazing, shrub lands, and forest area and an increase in cultivated land. This study's investigation of LULC change during 40 years between 1983 and 2022 revealed an increase in cultivated land of 47 541 km^2^. In contrast, shrublands, grazing land, and forests decreased by 31 664, 8630, and 10 139 km^2^, respectively. Furthermore, the built‐up area and the bare land area also increased by 1777.2 and 452 km^2^, respectively.

In line with this finding,^[^ ^96^ ^]^ indicated that cultivated land and built‐ups increased between 1957 and 1982 to 2246 ha (13% increase) but decreased by ≈586 ha (2%) between 1982 and 1998 in the Chemoga watershed in the Blue Nile basin. The expansion between 1957 and 1982 parallels population growth.^[^ ^54^, ^96^ ^]^ also reported that built‐ups and cultivated areas showed an increasing trend of 13 and 9.1%, respectively, while grazing lands, bare lands, and forest showed a decreasing trend of 10%, 9.8%, and, 2.3% respectively, in the Lake Tana basin of the UBNB. According to^[^ ^59^ ^]^ almost the entire highland area is farmland (33.9%). Further, forest, grasslands, woodlands, and shrublands occupied 1.41%, 23.1%, 20.3%, and 10.3% of the Abay Basin area, respectively.

In general, many previous studies indicated that agricultural lands and settlements have increased while grazing lands, shrublands, and forests have declined.^[^ ^6^, ^52^, ^54^, ^56^, ^83^, ^96^ ^]^ Hence, the expansion of cultivated and settlement areas is mainly due to the rapid population increase in the basin. The expansion of cultivated lands at the expense of grazing lands, shrublands, and forest areas has significant impacts on achieving sustainable development goals. Hence, it imposes on and affects the hydrological processes of the basin, escalating soil erosion and accelerating land degradation and deforestation in the basin. In general, the ecosystem services of the basin are significantly affected by the expansion of agricultural lands.

## 5 Conclusion

Random forest classification is an innovative machine‐learning technique that utilizes multiple decision trees to classify data. It is an efficient and accurate way of classifying data, and it is widely used in many fields. Additionally, random forest classification is more robust to outliers than traditional parametric classification techniques. The results indicated that the random forest classifier can achieve a classification accuracy that is better than that achieved by maximum likelihood and support vector machine classification algorithms. Ensemble‐based random forest algorithms hold the potential to transform difficult computational problems and uncover new solutions to address land use and cover pattern classification challenges. Hence, random forest‐based LULC classification has the highest accuracy as compared to conventional parametric statistical techniques of land use and land cover classification. This will help to create a deeper understanding of land use/cover change and its impacts on the environment. In addition, it will enable better decision‐making for sustainable development.

LULC change is analyzed for three time periods in the upper BNRB using Landsat images from 1983, 2003, and 2022. Cultivated lands, bare lands, built‐ups, grazing land, shrublands, and water bodies were the major LULC classes identified in the studied basin. In the basin, cultivated lands and built‐up areas significantly increased at the expense of grazing lands, shrub lands, and forest lands from 1983 to 2022 because of rapid population growth. On the other hand, water bodies in the basin also increased from 1983 to 2022 due to the construction of reservoirs such as small and large dams for irrigation purposes and the Grand Ethiopian Renaissance Dam for hydroelectric power generation.

The main determining factors behind cultivated land expansion are related to an increase in demand for farming lands due to population growth. Population growth also leads to forest lands conversion to farmlands and forests for fuel. In addition, construction activities would increase, which would cause the cutting of trees and the conversion of these lands into cultivated land. This would enable us to meet food demand. Furthermore, cultivation of shallow soils on steep slopes could lead to erosion of topsoils, sedimentation of water reservoirs, and overexploitation of forest resources. To manage natural resources effectively, conservation measures, such as afforestation and reforestation, are a necessity. Therefore, environmental rehabilitation should be improved by raising the community's understanding of the optimal use of natural resources, conservation practices, and their benefits. The study concluded that random forest classification from multispectral satellite imagery is a powerful and cost‐effective tool for land cover and land use classification. This technique can help us quickly and accurately map areas for conservation and land use planning. It can also be used to monitor land degradation and assess the impact of climate change.

## Conflict of Interest

The authors declare no conflict of interest.

## Data Availability

The data that support the findings of this study are available from the corresponding author upon reasonable request.
